# Tablet-Based Telerehabilitation Versus Conventional Face-to-Face Rehabilitation After Cochlear Implantation: Prospective Intervention Pilot Study

**DOI:** 10.2196/20405

**Published:** 2021-03-12

**Authors:** Christiane Völter, Carolin Stöckmann, Christiane Schirmer, Stefan Dazert

**Affiliations:** 1 Department of Otorhinolaryngology, Head and Neck Surgery Ruhr University Bochum St Elisabeth Hospital Bochum Germany; 2 Kampmann Hearing Aid Acoustics Bochum Germany

**Keywords:** computer-based auditory training, correction of hearing impairment, cochlear implant, effectivity, intervention study, telerehabilitation, pandemic

## Abstract

**Background:**

Technologies allowing home-based rehabilitation may be a key means of saving financial resources while also facilitating people’s access to treatment. After cochlear implantation, auditory training is necessary for the brain to adapt to new auditory signals transmitted by the cochlear implant (CI). To date, auditory training is conducted in a face-to-face setting at a specialized center. However, because of the COVID-19 pandemic’s impact on health care, the need for new therapeutic settings has intensified.

**Objective:**

The aims of this study are to assess the feasibility of a novel teletherapeutic auditory rehabilitation platform in adult CI recipients and compare the clinical outcomes and economic benefits of this platform with those derived from conventional face-to-face rehabilitation settings in a clinic.

**Methods:**

In total, 20 experienced adult CI users with a mean age of 59.4 (SD 16.3) years participated in the study. They completed 3 weeks of standard (face-to-face) therapy, followed by 3 weeks of computer-based auditory training (CBAT) at home. Participants were assessed at three intervals: before face-to-face therapy, after face-to-face therapy, and after CBAT. The primary outcomes were speech understanding in quiet and noisy conditions. The secondary outcomes were the usability of the CBAT system, the participants’ subjective rating of their own listening abilities, and the time required for completing face-to-face and CBAT sessions for CI users and therapists.

**Results:**

Greater benefits were observed after CBAT than after standard therapy in nearly all speech outcome measures. Significant improvements were found in sentence comprehension in noise (*P=*.004), speech tracking (*P=*.004) and phoneme differentiation (vowels: *P=*.001; consonants: *P=*.02) after CBAT. Only speech tracking improved significantly after conventional therapy (*P=.*007). The program’s usability was judged to be high: only 2 of 20 participants could not imagine using the program without support. The different features of the training platform were rated as high. Cost analysis showed a cost difference in favor of CBAT: therapists spent 120 minutes per week face-to-face and 30 minutes per week on computer-based sessions. For CI users, attending standard therapy required an average of approximately 78 (SD 58.6) minutes of travel time per appointment.

**Conclusions:**

The proposed teletherapeutic approach for hearing rehabilitation enables good clinical outcomes while saving time for CI users and clinicians. The promising speech understanding results might be due to the high satisfaction of users with the CBAT program. Teletherapy might offer a cost-effective solution to address the lack of human resources in health care as well as the global challenge of current or future pandemics.

## Introduction

### Background

In recent years, information technology solutions have been developed that allow professionals to treat patients via teletherapy. With regard to rapidly increasing health care expenses owing to the aging of society and even faster medical and technical advances, cost-effective rehabilitation is both a priority and a challenge for users and therapists [1]. This phenomenon has been stressed by the current COVID-19 pandemic crisis, which is transforming our society and has implications for health care [2-4]. Telemedicine has been shown to be an option in previous outbreaks, such as severe acute respiratory syndrome–associated coronavirus or Middle East respiratory syndrome coronavirus [4,5]. An additional benefit is that these digital solutions have the potential to reduce health care costs associated with supervision and high-frequency training [6-8].

So far a teletherapeutic approach is often used in psychotherapeutic sessions with a high level of satisfaction and compliance [9-11]. A positive outcome after home-based therapy has also been reported in patients with chronic pain [12] and those who received knee or hip replacements [13].

### Rehabilitation After Cochlear Implantation

Auditory training is an important part of rehabilitation after cochlear implantation. Several consensus papers have reported that it is necessary for the brain to adapt to the new auditory stimulus transmitted by the implant [14-16]. However, rehabilitation after cochlear implantation differs among countries. In some countries, postoperative rehabilitation programs are not routinely offered because of a lack of reimbursement by health insurance companies and a shortage of specialized therapists [17], whereas in others, cochlear implant (CI) recipients follow an intensive rehabilitation regime that is regularly covered by the general health insurance for at least 2 years after surgery [16]. Auditory training usually takes place in a face-to-face setting in specialized centers; computer-based applications are used only as an additive to standard (face-to-face) therapy. In a previous study, we developed Train2hear, which is a highly individualized digital training platform that combines different components of adaptivity, feedback, and motivation to allow CI users to receive computer-based auditory training (CBAT) that is tailored to their specific therapeutic needs. The first evaluation, within the setting of an applicant’s workshop, clearly demonstrated that CI users enjoyed using Train2hear [18]. A challenge faced by teletherapy is to achieve the same efficiency as standard face-to-face therapy.

### Computer-Based Auditory Training

Few studies have assessed the effectiveness of digital auditory rehabilitation in adult CI users, and these studies also have only analyzed some aspects in a small number of participants (ie, less than 20) [19-21]. In Schumann et al [22] 15 CI users received 3 weeks of training on phoneme discrimination. A control group and follow-up assessments were not included. Fu et al [23] used a similar approach and studied phoneme discrimination in 10 participants over 4 weeks. In addition to improved performance in trained skills, a transfer effect on sentence comprehension was observed. This observation contrasts with that of Stacey et al [24] who found a significant improvement in consonant discrimination but not in sentence comprehension. Self-perceived improvement was reported in only 2 of the 11 participants. The only publication so far that has compared standard face-to-face therapy with a computer-based approach was by Bernstein et al [6] who analyzed speech tracking ability in 9 patients after a 4-week period. In their study, the tracking rate was improved, but no difference was observed between the two methods.

Furthermore, only a few studies have investigated the ability to listen in noise after CBAT [19,21,25]. However, there were conflicting results, with small number of participants. In Ingvalson et al [21], 5 CI users with postlingual deafness and at least one year of hearing experience showed improved speech perception only in quiet conditions. In contrast, Oba et al [19] reported a significant improvement in babble and steady noise after a 4-week digit training in 10 participants with CI. Even Green et al [25] observed in 9 participants with postlingual deafness that the thresholds to understand 50% of the sentences presented in noise significantly improved after 4 weeks of training in noise, but transfer effects on phoneme discrimination and memorization could not be demonstrated. In short, a systematic evaluation of a complete teletherapeutic rehabilitation program is lacking.

Therefore, the aims of this study are (1) to assess the usability and feasibility of the CBAT platform Train2hear in adult CI users; (2) study the objective and subjective auditory development as well as the economic benefit after a 3-week tablet-based rehabilitation as compared with a 3-week conventional face-to-face setting; and (3) analyze the impact of sociodemographic variables on outcomes.

## Methods

### Participants

In total, 20 adult CI users were included in this study (Table 1). To be included in the study, potential participants had to be adults (≥18 years); CI users with postlingual bilateral hearing loss and a CI experience of at least 3 months; have no significant motor, visual, or cognitive impairment; be willing and able to complete the tasks inherent in the study; and to give their informed consent. All subjects attended weekly face-to-face therapy at the implant center before the study (range: 7-48 sessions; SD 10.3).

**Table 1:** Profile of the participants (n=20).

Characteristics		Value
**Age (years)**		
	Mean (SD)	59.4 (16.3)
	Range	26-82
**Sex, n**		
	Female	14
	Male	6
**Years of education**		
	Mean (SD)	11.8 (1.7)
	Range	8-17
**Duration of hearing impairment (years)**		
	Mean (SD)	29.4 (19.9)
	Range	1-74
**Hearing loss in contralateral ear (dB)**		
	Mean (SD)	78.6 (27.3)
	Range	22.5-120
**Cochlear implant experience (months)**		
	Mean (SD)	10.3 (5.3)
	Range	3-22
**Etiologies of hearing loss, n**		
	Idiopathic sudden hearing loss	6
	Viral infection	4
	Meniere disease	3
	Chemotherapy	2
	Petrous bone fracture, cholesteatoma	2
	Unknown cause	2
	Acoustic trauma	1

### Economic Evaluation

During the intervention period, costs were measured for both CBAT and face-to-face therapy according to the international guidelines for conduction cost analysis [26,27]. Cost-related data covering costs relevant to the health center and costs for the patients were assessed on a standardized cost sheet for each patient. Subsequently, the costs of the two treatment modalities were compared.

### Study Design

All participants performed at least seven therapeutic face-to-face sessions in the rehabilitation center before the start of the study (mean 26.3, SD 10.3). Internet access and an audio loop were required to use the telerehabilitation system at home. The tablets were provided by the clinic.

**Figure 1:**
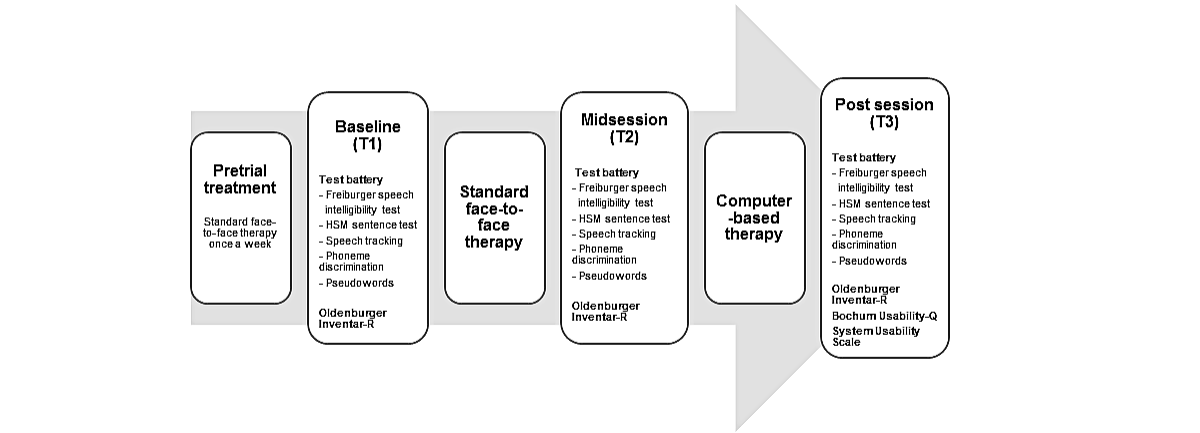
Timeline of the study.

Participants completed 3 weeks of conventional face-to-face rehabilitation followed by 3 weeks of self-training with the home-based digital auditory training program, Train2hear. All participants were assessed at baseline, after the 3-week face-to-face rehabilitation, and after the 3-week digital training program, as shown in Figure 1.

### Outcome Assessment

#### Freiburg Speech Intelligibility Test

Speech comprehension on word level in quiet was examined using the Freiburg Speech Intelligibility Test [28]. In total, 20 monosyllabic words and 10 two-digit numbers were presented to the participants in free field at 65 dB. For each test session, different but comparable lists were chosen to prevent false learning effects. Lists 1, 3, and 5 for the number test and lists 6, 7, and 5 for the monosyllabic test were chosen. For participants with residual hearing in the contralateral ear, masking was performed with an earplug and acoustic earmuffs.

#### Hochmair-Schulz-Moser Sentence Test

Speech perception of sentences in noise was measured by the Hochmair-Schulz-Moser (HSM) sentence test, which contains 3 exercise lists and 30 test lists with 20 sentences of everyday life [29]. Different comparable test lists (lists 5, 6, and 7) were presented at 65 dB with a signal-to-noise ratio (SNR) of +10 dB.

#### Speech Tracking

Speech tracking, as described by Filippo and Scott [30], was assessed using SpeechTrax, developed by MED-EL (Innsbruck). Over a period of 5 minutes, the short story *The lighter* by Hans Christian Anderson was presented via a live voice by an experienced speech and language pathologist with 70 words per minute. Participants were asked to repeat word by word and sentence by sentence. Afterwards, the tracking rate was calculated by dividing the total number of words the patient understood by the duration of the test. For participants with residual hearing in the contralateral ear, masking was performed using an earplug and acoustic earmuff.

#### Phoneme Discrimination

Phoneme discrimination was tested by presenting 7 vowels (a, e, i, o, u, ü, and ö) and 16 consonants (d, t, k, g, w, f, ch, sch, r, l, b, p, n, m, s, and z). Presentation was performed via an audio file and an audio loop. The consonants and vowels were presented in nonsense syllables (vowels:/m/-vowel-/m/; consonants:/a/-consonant-/a/), as described by Schumann et al [22]. The participants were asked to choose the target item from a selection of distractors. For vowels, all other target items were used as distractors (n=7). For consonants, the distractors were selected based on the similarity of the articulation´s location, type of articulation, and pitch. Items were presented in a random order to avoid the learning effect.

#### Pseudowords

To evaluate auditory perception independent of cognition and linguistic competence, pseudowords (30 nonwords with a length of 2-6 syllables) from the Mottier test were presented via an audio loop [31]. The participants were required to repeat the words as accurately as possible. In the first step, the ability to determine the number of syllables in the target word was analyzed. In the second step, the number of correctly repeated syllables was counted.

#### System Usability Scale

Train2hear’s usability was assessed using the System Usability Scale (SUS) questionnaire [31]. The SUS comprises 10 questions, each answerable on a 5-point Likert scale in which the end points are *I strongly disagree* and *I strongly agree*. For the 5 statements in which *I strongly agree* is a positive assessment of Train2hear, an answer of *I strongly agree* is worth 4 points and an answer of *I strongly disagree* is worth 0 points. This scoring method is reversed in the 5 statements in which *I strongly agree* would be a negative assessment of the Train2hear. Thus, the higher the score, the more positive is the assessment. A score of >68 indicates a high level of usability [32].

#### Bochum Usability Questionnaire

A specific questionnaire was developed with 34 closed questions covering 8 aspects of Train2hear’s training platform: (1) implementation of the program, (2) exercises, (3) feedback, (4) statistics, (5) handling regarding videoconferencing, (6) design, (7) motivational elements, and (8) overall assessment of the training program. Participants answered on a Likert scale from 0 to 4, with higher scores indicating better results. The total score for each subtest and each individual question was assessed.

#### The Oldenburger Inventory-R Score

Participants evaluated their own auditory skills based on the Oldenburger Inventory-R questionnaire [33], which assesses hearing in everyday situations. The 32 closed questions were divided into 7 categories: hearing in silent and in noisy conditions, localization, hearing effort, social interaction, and listening abilities. The subtest entitled *Other* includes questions about discrimination and perception of sounds, voices, and music. For all categories except *social interaction*, higher scores indicate a better subjective perception of hearing status. Multimedia Appendix 1 shows an English translation of the questions and categories.

### Auditory Training

#### Face-to-Face Training

After baseline testing, all 20 participants received face-to-face therapy (120 minutes each, once a week for 3 weeks) according to the regular rehabilitation schedule by an experienced speech and language pathologist at the CI center. The content of the sessions was tailored to the participants’ needs as assessed at the baseline assessment and according to a rehabilitation concept that is in accordance with (1) the guidelines of the German Society for Otorhinolaryngology, Head and Neck Surgery and (2) the current concepts of speech processing and auditory rehabilitation [34,35].

Therapists selected exercises on different auditory levels (detection, discrimination, identification, and understanding of syllables, words, sentences, and complex speech) and applied a synthetic and analytic approach. Tasks on word, sentence, and text comprehension were presented in closed or open sets with or without background noise (different SNRs) in live and computerized voices.

#### CBAT

Train2hear was based on a previously developed auditory training program for adult CI users [18]. The platform incorporates (1) an initial evaluation of the user´s body functions and structures, participation, and hearing status according to the International Classification of Functioning, Disability and Health and (2) an automatic adaptation of the exercises to the participant’s performance.

Participants performed 27 exercises in total, which were arranged in a hierarchical order as supposed by the hearing model of Erber [34]. They started with the simplest exercises such as tasks on sound differentiation and identification to the most difficult ones, such as speech understanding in noise. Background noise varied between 15 dB and 5 dB SNR, depending on the patient's auditory abilities.

**Figure 2:**
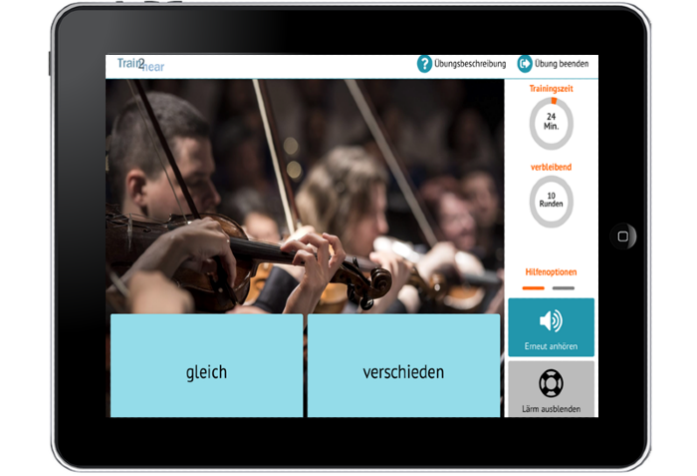
Example of an exercise (differentiation of different instruments, gleich in German means similar, verschieden in German means different).

Screenshots illustrating different parts of the Train2hear intervention are presented in Figure 2 and Multimedia Appendices 2 and 3. For more detailed information, please see a recent publication by our group, where a specific description of the different exercises as well as the motivation and feedback mechanisms is included [18].

Before starting the Train2hear training, all participants were shown by an experienced speech and language pathologist on how to use the system. The participants then independently performed CBAT 5 days per week for 25 minutes each. Subjects with residual hearing in the contralateral ear were trained only with the implanted ear using an audio loop. After 10 days, a videoconference chat between the participants and the language therapist took place to check the participants’ adaptation, review the program, and assess adverse events.

### Statistical Analysis

First, a descriptive analysis of the data using the mean value and SD was performed.

Thereafter, rank-analysis of variance (ANOVA; Friedman test) was performed to prove that there were significant changes between the three measurements (T1, T2, and T3). Afterward, the Wilcoxon signed-rank test for paired samples was applied to evaluate participants’ results after the two types of therapy. For rank binding, a sign test was applied. The exact *U* test was used to determine the correlation between the results and sex.

Further correlations between outcome and continuous sociodemographic factors, such as age and years of education, were calculated. If there was no normal distribution, then the Kendall rank correlation in case of rank binding was applied. The significance level was set at *P=.*05. Statistical analyses were performed using Medas (Grund).

Ethics committee approval (19-6618-BR) was received from the Ethics Committee of the Medical Faculty of Ruhr University Bochum.

## Results

### Therapy Time

All participants completed face-to-face and CBAT training sessions. For each participant, therapists spent 360 minutes (3×120 minutes) on face-to-face procedures and 60 minutes (30 minutes of videoconferencing and 30 minutes of introducing the digital program) for CBAT. To participate in the 3-week face-to-face training, participants needed approximately 6 hours of travel time (range: 1.5-12 hours).

Previous experience of using digital media differed among the participants: 13 had regularly used a computer (daily or several times a week), whereas 4 had never worked with a computer. In total, 11 participants regularly used a tablet several times a week, whereas three did not. All other participants had previous experience of using digital devices (tablets, smartphones, and computers). Before the study, 16 participants had no experience with videoconferencing, whereas 4 had used videoconferencing to communicate with family members or friends.

Digital experience did not correlate with speech understanding assessed by the test battery at T2 or T3. In contrast, affinity to digital media had a significant impact on the assessment of the usability of the program. Participants who frequently used a computer stated significantly more often that the videoconference was easy to use (*P=*.03; Bochum Usability Questionnaire Q16). A significant positive correlation among questions 3, 5, 6, and 7 of the SUS and digital experience could be detected. Participants with more experience judged the program to be easier to use (*P=*.03; SUS Q3) and more often stated that the different functions were well integrated (*P=*.02; SUS Q5). Experienced users also stated, significantly more often, that the handling of the program could be learned quickly (*P=*.007; SUS Q7). Nevertheless, regular tablet users still found the program cumbersome to use (*P=*.02; SUS Q8).

### Test Outcome

The results of the test battery at baseline (T1), after face-to-face therapy (T2), and after CBAT (T3) are shown in Multimedia Appendix 4. Tests that did not significantly differ in rank-ANOVA (Friedman test) between the three test times were not further investigated (Multimedia Appendices 4 and 5).

#### Freiburg Speech Intelligibility Test

Neither the Freiburg number test nor the Freiburg monosyllabic test showed significant changes during the study. However, the following correlations between test performance and sociodemographic data could be identified: regarding monosyllabic speech comprehension, older participants were less likely to benefit (*P=*.04) from the CBAT. At T3, the results depended on sex (*P=*.04): men’s score was increased by 19.2%, whereas women’s score was slightly decreased by 1.1%. In addition, participants with more hearing experience showed less improvement (*P=.*04) after the CBAT. At the end of the intervention (T3), prolonged hearing impairment was negatively related to performance in the Freiburg monosyllabic test (*P=*.04) and the Freiburg number test (*P=*.004).

#### HSM Sentence Test

The mean HSM scores improved significantly from T2 to T3 (*P=*.004). At the last assessment (T3), the duration of hearing loss and improvement were significantly correlated (*P=*.02). Age and sex did not affect the results either at T2 or T3 (age, *P=*.39; sex, *P=*.90), but the performance in the Freiburg monosyllabic test was significantly associated with the improvement in sentence comprehension at T3 (*P=*.04). Furthermore, participants with better results in the HSM rated their ability to understand speech in noise significantly better, as shown in the Oldenburger Inventar-R Questionnaire (*Listening in noise*) subscore (*P=*.03).

#### Speech Tracking

The speech tracking rate significantly increased. At T1, participants had a tracking rate of 31.3 words per minute (wpm; SD 16.38), which increased after face-to-face training by 4.92 wpm (SD 7.26; *P=*.009). After 3 weeks of CBAT, the subjects reached 41.3 wpm (SD 18.29; *P=*.003). Sex, age, hearing experience, and duration of hearing loss had no impact on performance. Monosyllabic word recognition (Freiburg) at baseline was significantly correlated with improvement in speech tracking at T2 (*P=*.02).

#### Phoneme Discrimination

Comparing T2 and T3, improvements in vowel discrimination (*P=*.001) and consonant discrimination (*P=*.02) were observed. A shorter duration of hearing loss was significantly correlated with an improvement in vowel discrimination between T2 and T3 (*P=*.02).

The ability to discriminate consonants was also significantly associated with age and the duration of hearing loss (T1-T2). Older participants (*P=*.02) and participants with hearing loss for a longer period showed less improvement (*P=*.03).

#### Pseudowords

Throughout the study, no significant changes were observed in the identification of syllables (*P=*.64) and the repetition of syllables (*P=*.51). These observations did not depend on the length of the items. The results indicated that participants with better monosyllabic comprehension at T1 were able to repeat the syllables more accurately (*P=*.009).

#### SUS

Participants evaluated the usability of the Train2hear program as excellent (mean score: 87.0; SD 12.1; Multimedia Appendix 6). Question 1 received one of the highest scores: 18 out of 20 participants stated that they could imagine using the program regularly. In total, 70% (14/20) of the participants indicated that the various functions were well integrated into the program (Q3), 100% (20/20) agreed that the program was easy to use (Q2), and 95% (19/20) felt confident using the program (Q5).

The only questions with lower scores (Q4, Q7, and Q10) referred to the handling of the technology and the support necessary at the beginning of the training. No additional support was necessary in 70% (14/20) of cases (Q7).

The structure of the program (Q6, Q8, and Q9) was judged to be good. Only one participant claimed the program to be too complex (Q6) and too cumbersome to use (Q9). Two patients judged the program to be inconsistent. A significant correlation was found between questions Q4 (need for support) and Q10 (need for guidance) and age, both of which were rated worse by older participants (Q4, *P=*.008; Q10, *P=*.007). The overall score was also age-related: older participants were more critical than younger participants (*P=*.006; Multimedia Appendix 6).

#### Bochum Usability Questionnaire

The overall design of the program was rated very good. In total, 100% (20/20) liked the design (Q19), and the font size (Q20) and buttons (Q21) were judged to be appropriately sized by all the participants.

More than 60% of the participants rated the training tips and introduction videos to be very helpful (Q1, Q2). The participants liked the concept of a journey through Europe (Q9, mean 97.5%, SD 0.45). In this context, 95% (19/20) of the participants considered the tasks to be relevant to everyday life (Q3). The level of the exercises was appropriate for 17 out of 20 participants. All the questions concerning the exercises reached 89% (71.4/80) of the maximum score. The program statistics were regularly used by 70% (14/20) at least once a week (Q13). Presentation of the statistical data was comprehensible for 85% (17/20) (Q15). However, 20% (4/20) of the subjects declared that statistics did not help them to better understand their results. In general, the statistical features were rated as the weakest of all categories. The score reached 75% (60/80) of the maximum score.

Most participants would recommend the program to others (Q30, mean 98.8%, SD 0.23). An obligatory training time of 25 minutes per day could be conducted by the majority (Q34, mean 98.8%, SD 0.23). Older participants stated more frequently that CBAT could be an addition to face-to-face training (Q29, *P=*.007) and that working with Train2hear was highly motivating (Q31, *P=*.001). In addition, they had fewer problems conducting dedicated training days per week (Q33, *P=*.04).

Female participants judged the feedback to be significantly better than male participants (Q11, *P=*.04). However, there was no sex-related difference in the enjoyment of training (Q22, *P=*.08). Participants with a higher educational level would recommend computer-based training to others more frequently (Q30, *P=*.01) and were more satisfied with the support provided (Q8, *P=*.04). Furthermore, they claimed that videoconferencing was as satisfying as personal contact (Q18, *P=*.04).

The most significant correlation was found between the years of education and questions related to technology. Participants with higher education reported more often that the technology worked without any problems (Q26, *P<*.001). Participants with less education judged the program's feedback to be significantly better (Q10, *P=*.01). Participants with a lower speech perception score at T2 (as assessed by the Freiburg Speech Intelligibility Test) were more likely to feel anxious while using the program (Q25, *P=*.01; Multimedia Appendix 7).

#### Oldenburger Inventory Score

The subjects’ self-perception did not change significantly in this study. As shown in Multimedia Appendix 8 this refers to all subcategories except for listening in noise, which has been judged to be better after face-to-face therapy (*P=*.003). Sociodemographic variables affected localization abilities, social interaction, listening effort, and the development of auditory skills in general.

Localization abilities were related to sex. Comparing T2 and T3 women achieved significantly worse results than men (*P=*.03). A correlation with sex was also evident in the social interaction subscale (*P=.*04). Furthermore, there was an association between social interactions and age. Younger participants improved significantly more due to CBAT (T2-T3; *P=.*04). Furthermore, age was negatively related to the development of auditory skills at T3 (*P=.*008). With regard to the duration of hearing loss, a negative correlation with listening effort was detected at T2 (*P*=.001).

### Economic Evaluation

To attend the face-to-face session, patients had to travel 237 km (SD 80.7), which entailed spending on an average of 234 minutes (SD 58.6) on the road. Therapists devoted 450 minutes for a standard face-to-face therapy and 90 minutes per patient for CBAT (including the time of preparation and documentation). Therefore, costs could be reduced from €262.50 (US $320.25) to €52.50 (US $64.05) for the study period (Multimedia Appendix 9).

If standard face-to-face therapy, which regularly included 20 sessions of speech therapy (each of which lasted 120 minutes) was completely replaced by CBAT, then the costs would decrease from €1750.00 (US $2134.00) to €350.00 (US $427.00) based on the data obtained in this pilot study.

## Discussion

### Principal Findings

This study is, to the best of our knowledge, one of the first to demonstrate that a digital auditory rehabilitation program might reduce adult CI users’ dependence on human resources while ensuring that they receive a clinical outcome similar to that of standard therapy, that is, conventional face-to-face rehabilitation at a specialized rehabilitation center.

A comparison of the two auditory training methods (face-to-face and CBAT) revealed a greater benefit in sentence comprehension in background noise after CBAT. This may be explained by the application of the training schedule. Teletherapeutic tasks were performed five to seven times a week, whereas outpatient therapy was performed only once a week. This is in line with Vu et al [36] who found significant differences in log-in frequency and learning activities between successful and unsuccessful learners in web-based training for teachers. The most remarkable improvements were detected in phoneme discrimination and speech tracking, which are closely related to interactive communication [6,37].

Overall, the Train2hear program was rated as highly usable by the participants. The fact that older participants rated usability worse than younger participants may be related to the lower level of technical experience among older people. This result was also mentioned by Ferguson and Henshaw [38], who stated that access to hardware and lack of skills in using hardware hinder access to computer-based training. Regardless of this age-related difference, all participants agreed that Train2hear is easy to use. Nonetheless, external support may be helpful for older users. This could be done either by the user’s partner or family or friends or by therapists via videoconferencing.

In contrast, older participants had significantly higher scores on motivation and ease of adherence to the training schedule than did younger participants. In general, age did not significantly influence performance; the Freiburg monosyllabic test was the only speech understanding test in which older participants scored worse than their younger counterparts as compared with face-to-face therapy and CBAT (*P=*.004). Prolonged training intervals might have a positive effect because of a slower learning curve in older adults [6].

Nonusage has been known to be an important barrier in the field of web-based training [39], especially in intervention*s* using automatic functions with minimal human involvement. In this study, 100% (20/20) of the participants completed the 3-week digital training program. As compared with other studies, this adherence rate can be interpreted as extraordinarily high [39].

A possible explanation for the high adherence rate could be that the Train2hear software is highly individualized, which includes a basic assessment of the user’s demands and needs and automatically adapts the training schedule to their performance.

Furthermore, the Train2hear platform contains various motivational elements that might lead to better user adherence, for example, a close feedback system and reminders [38-40]. However, it remains to be seen if such levels of adherence would continue at a long-term follow-up. In a study on patients with stroke, Jurkiewicz et al [41] found that adherence in the initial period was significantly higher than that in the long-term follow-up. Previous works have shown that incorporating an avatar can increase motivation and engagement with a training application and the time spent in training [24,42,43]. With this observation in mind, we added a train conductor as an avatar to the new training platform.

Educational level had a significant impact on the handling of the software. This result is in line with Kriwy and Glöckner, who reported that the higher an individual’s level of education, the better they could take part in computerized health programs [44].

Furthermore, significant correlations were observed between the total duration of hearing loss and improvements after T2. Generally, the shorter the duration of hearing loss, the greater the improvement in speech understanding. This result was also assumed by Ihler et al [45] in their study on home-based auditory training of speech recognition on the telephone in 20 CI users with postlingual hearing loss. Whether auditory training over a longer period can lead to greater improvements in speech comprehension, even in people with a long duration of hearing loss, has yet to be proven.

Participants’ self-evaluated hearing abilities remained nearly unchanged after both face-to-face training and CBAT. Previous studies have reported this result. No, or only minor, self-reported improvements of listening abilities after auditory training periods have also been reported by Stacey et al [24] (after 5 days a week for 3 weeks) and Bernstein et al [6] (once a week for 8 weeks). The question is, if despite objectively shown improvements in speech understanding, a training period of 3 weeks is too short to have an impact on self-perceived hearing status.

There is currently an acute need to study the effectiveness of therapeutic interventions in speech language pathology and audiology. Studies designed and conducted in accordance with evidence-based criteria provide a rational basis for therapeutic approaches that are missing in large parts of auditory therapy [46]. CBAT might be an appropriate tool for future multicenter studies because the protocol is well defined (although highly individualized) and therefore comparable. In addition, CBAT enables a large amount of data to be obtained during the entire training procedure. This process can help speech and language pathologists to more precisely investigate the progress of CI users and to evaluate and refine the therapeutic approach.

Regarding the time- and resource-saving potential of CBAT, each therapist saved more than 5 hours per participant during the 3-week training period, including the time they would have needed to prepare the lessons. The participants saved a mean of almost 4 hours of traveling. Regarding the intense rehabilitation program that is regularly offered to CI recipients in Germany for 2 years and reimbursed by the general health insurance, home-based training might save an enormous amount of economic and human resources even if it might be suitable only for selected CI users and limited to only some parts of the rehabilitation process.

Furthermore, at the time of writing, much of the world is under lockdown or some form of restriction due to the COVID-19 pandemic. In such situations, people are not able to access office-based therapy; therefore, CBAT might be an appropriate and crucial tool for successful hearing rehabilitation, especially for older CI users and those with weakened immune systems.

### Previous Studies

The few studies that exist on CBAT have a limited scope. They evaluated only a few aspects of auditory training [6,22,45] and generally had short or no follow-up assessments [19,23,25]. Previous studies have described improvements in speech comprehension and communication skills after several weeks of CBAT [20,47,48].

Overall, studies usually included only a small sample size [47]. Only Bernstein et al [6] compared the standard face-to-face regimen with digital auditory training. They conducted their study on speech tracking performance in CI users. Similar to our results, they found that CI users had an improved tracking rate (*P*<.001) and sentence recognition (*P*<.001) using both therapeutic approaches.

### Limitations

Although this study is one of the largest on CBAT in terms of the number of participants, 20 participants were still a limited study group. In our study, we chose only a period of 3 weeks because of the regulations of the research project. It must be kept in mind that this period is short compared with the long rehabilitation period of 2 years, which is regularly performed in Germany after cochlear implantation. Furthermore, future studies would benefit from increasing the duration of the training period and analyzing the long-term effects to better evaluate how effective CBAT is and how well users adhere to the training program.

Due to the study design, it cannot be completely ruled out that the positive outcome after CBAT is partially due to the long-term effects of the conventional face-to-face training sessions. However, all participants had experience with face-to-face therapy before the study. This is a bias that all therapeutic studies are faced with. A complete stop of the training over a longer period would be necessary to rule out long-term effects, and this is not ethically justifiable.

Even the inclusion of a control group could not have solved this problem because CI recipients widely differ in terms of age, duration of hearing loss, socioeconomic status, etc. Therefore, we cannot completely rule out the effects of age, sex, duration of hearing loss, technical experience, and hearing experience on treatment outcomes. However, these correlations did not show a significant association. Large multicenter studies should be conducted in the near future to confirm the presented data.

### Conclusions

Due to global demographic changes and the pressure under the current COVID-19 pandemic, there is an enormous and increased need for computerized therapeutic interventions in speech language pathology and audiology. Computer-based auditory therapy is an evidence-based and standardized yet highly individualized approach that has the potential to save human and economic resources. Outcomes seem to be quite similar to face-to-face therapy although due to the small number of participants, the results have to be confirmed. However, the promising results of this pilot study justify further investigation and evaluation of the Train2hear program in a large multicenter study over a longer period.

## Appendix

Multimedia Appendix 1 Subjective audiological self-rating based on Oldenburger Inventory-R score (Audiologie Initiative Niedersachsen, 2007).

Multimedia Appendix 2 Overview of exercises performed in Vienna (for example users had to count syllables and to differentiate vowels on a sightseening tour through Vienna).

Multimedia Appendix 3 Training schedule and calendar.

Multimedia Appendix 4 Results of each test at T1, T2 and T3 in n=20 (100%).

Multimedia Appendix 5 Test battery rank–ANOVA (analysis of variance).

Multimedia Appendix 6 System Usability Scale (Brooke, 1996); n=20 (100%) for each statement.

Multimedia Appendix 7 Bochum Usability Questionnaire; n=20 (100%) for each statement except in subtest “Videoconferencing” n=15 (100%).

Multimedia Appendix 8 Results of subjective audiological self-rating based on the Oldenburg Inventory-R score; n=20 (100%) for each test and interval.

Multimedia Appendix 9 Overview of the costs during the 3-week intervention study. (A) Patient’s data. (B) Therapist’s data.

## Abbreviations

CBAT: computer-based auditory training
CI: cochlear implant
EFRE: Europäische Fonds für Regionale Entwicklung
HSM: Hochmair-Schulz-Moser
SNR: signal-to-noise ratio
SUS: System Usability Scale
