# Supplementary figures and images for: Tablet-Based Telerehabilitation Versus Conventional Face-to-Face Rehabilitation After Cochlear Implantation: Prospective Intervention Pilot Study
Source: JMIR Rehabil Assist Technol. 2021 Mar 12;8(1):e20405. doi: 10.2196/20405 (PMC8082947; doi:10.2196/20405)

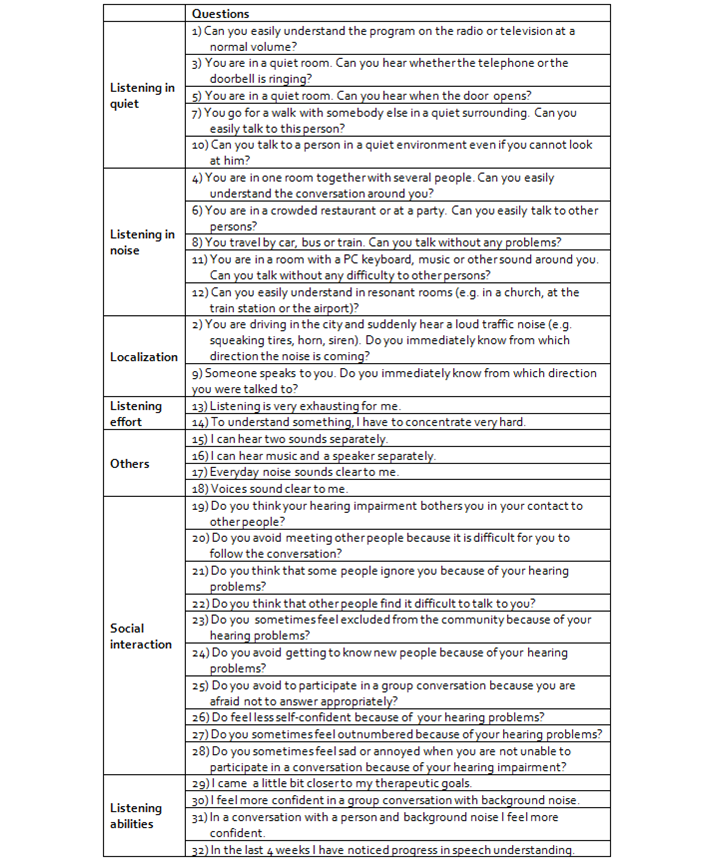

Supplement: Multimedia Appendix 1 [file rehab_v8i1e20405_app1.png]

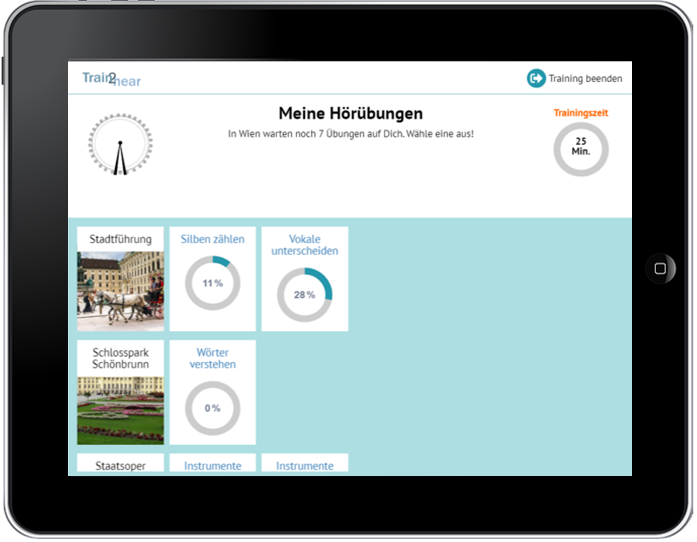

Supplement: Multimedia Appendix 2 [file rehab_v8i1e20405_app2.png]

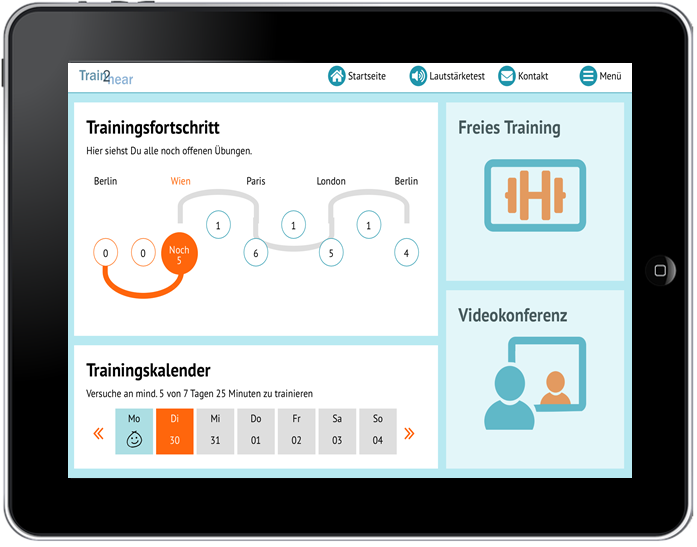

Supplement: Multimedia Appendix 3 [file rehab_v8i1e20405_app3.png]
