# Supplementary material for: Tablet-Based Telerehabilitation Versus Conventional Face-to-Face Rehabilitation After Cochlear Implantation: Prospective Intervention Pilot Study
Source: JMIR Rehabil Assist Technol. 2021 Mar 12;8(1):e20405. doi: 10.2196/20405 (PMC8082947; doi:10.2196/20405)
Supplement: Multimedia Appendix 4 [file rehab_v8i1e20405_app4.docx]

**Multimedia Appendix 4.** Results of each test at T1, T2 and T3 in n=20 (100%).

| **Test** | **time of testing** | **mean** | **SD** | **P** |
| --- | --- | --- | --- | --- |
| **Freiburger monosyllabic test** | T1 | 37.0 % | 24.4 | .68 |
|  | T2 | 35.8 % | 23.3 |  |
|  | T2 | 35.8 % | 23.3 | .15 |
|  | T3 | 40.8 % | 23.8 |  |
| **Freiburger number test** | T1 | 78.0 % | 21.7 | .77 |
|  | T2 | 78.0 % | 23.1 |  |
|  | T2 | 78.0 % | 23.1 | .34 |
|  | T3 | 83.0 % | 15.9 |  |
| **HSM sentence test** | T1 | 32.4 % | 31.1 | .55 |
|  | T2 | 31.7 % | 30.5 |  |
|  | T2 | 31.7 % | 30.5 | .0036** |
|  | T3 | 40.4 % | 32.7 |  |
| **Speech Tracking Rate (STR)** | T1 | 31.3 wpm | 16.4 | .0095** |
|  | T2 | 36.2 wpm | 18.4 |  |
|  | T2 | 36.2 wpm | 18.4 | .0036** |
|  | T3 | 41.3 wpm | 18.3 |  |
| **Vowel Differentiation** | T1 | 2.1/7 | 1.3 | 0.79 |
|  | T2 | 2.2/7 | 1.4 |  |
|  | T2 | 2.2/7 | 1.4 | .0023** |
|  | T3 | 3.1/7 | 1.4 |  |
| **Consonant Differentiation** | T1 | 9.8/16 | 2.9 | .79 |
|  | T2 | 10.1/16 | 3.1 |  |
|  | T2 | 10.1/16 | 3.1 | .023* |
|  | T3 | 11.4/16 | 2.2 |  |
| **Pseudowords**  Identification of syllables | T1 | 66.4% | 29.2 | 0.79 |
|  | T2 | 71.6% | 20.3 |  |
|  | T2 | 71.6% | 20.3 | 0.23 |
|  | T3 | 77.4% | 17.9 |  |
| Repetition of syllables | T1 | 13.1% | 18.2 | 0.063 |
|  | T2 | 16.9% | 19.0 |  |
|  | T2 | 16.9% | 19.0 | 0.12 |
|  | T3 | 18.2% | 16.7 |  |
