# Supplementary material for: Tablet-Based Telerehabilitation Versus Conventional Face-to-Face Rehabilitation After Cochlear Implantation: Prospective Intervention Pilot Study
Source: JMIR Rehabil Assist Technol. 2021 Mar 12;8(1):e20405. doi: 10.2196/20405 (PMC8082947; doi:10.2196/20405)
Supplement: Multimedia Appendix 5 [file rehab_v8i1e20405_app5.docx]

**Multimedia Appendix 5.** Test battery rank–ANOVA (analysis of variance).

| **Test** | **Friedman-Test (Rank-ANOVA)** |
| --- | --- |
| Freiburger monosyllablics | *.69* |
| Freiburger numbers | *.78* |
| HSM sentences | *.0045*** |
| Speech Tracking | *.00008**** |
| Vowel Differentiation | *.0035*** |
| Consonant Differentiation | *.047** |
| Pseudo-words | |
| *Identification of syllables* | *.38* |
| *Repetition of syllables* | *.029** |
| Oldenburger Inventory - R |  |
| *Listening in quiet* | *.084* |
| *Listening in noise* | *.028** |
| *Localization* | *.49* |
| *Listening effort* | *.74* |
| *Others* | *.54* |
| *Social interaction* | *.31* |
