# Supplementary material for: Tablet-Based Telerehabilitation Versus Conventional Face-to-Face Rehabilitation After Cochlear Implantation: Prospective Intervention Pilot Study
Source: JMIR Rehabil Assist Technol. 2021 Mar 12;8(1):e20405. doi: 10.2196/20405 (PMC8082947; doi:10.2196/20405)
Supplement: Multimedia Appendix 6 [file rehab_v8i1e20405_app6.docx]

**Multimedia Appendix 6.** System Usability Scale (Brooke, 1996); n=20 (100%) for each statement.

|  | Statement | I strongly disagree0 | 1 | 2 | 3 | I strongly agree4 | Total (%) | **SD** |
| --- | --- | --- | --- | --- | --- | --- | --- | --- |
| 1 | I think that I would like to use the program frequently. | 0 | 0 | 0 | 2 | 18 | 78 (97.5) | 0.31 |
| 2 | I think the program was easy to use. | 0 | 0 | 0 | 9 | 11 | 71 (88.8) | 0.51 |
| 3 | I think the various functions are well integrated into the program. | 0 | 0 | 0 | 6 | 14 | 74 (92.5) | 0.47 |
| 4 | I can imagine that most people will adapt to the programm quickly. | 0 | 0 | 4 | 4 | 12 | 68 (85.0) | 0.83 |
| 5 | I felt very confident using the program. | 0 | 0 | 1 | 4 | 15 | 74 (92.5) | 0.58 |
|  |  | I strongly disagree **4** | **3** | **2** | **1** | I strongly agree **0** | **Total (%)** | **SD** |
| 6 | I think the program is too complex. | 16 | 2 | 1 | 0 | 1 | 72 (90.0) | 0.99 |
| 7 | I needed support to use the program. | 9 | 5 | 1 | 3 | 2 | 56 (70.0) | 1.44 |
| 8 | I think there was too much inconsistency in the program. | 16 | 2 | 0 | 0 | 2 | 70 (87.5) | 1.24 |
| 9 | I think the program is very cumbersome to use. | 16 | 2 | 1 | 0 | 1 | 72 (90.0) | 1.00 |
| 10 | I had to learn a lot of things to be able to use the program. | 13 | 1 | 2 | 2 | 2 | 61 (76.3) | 1.47 |
