# Supplementary material for: Tablet-Based Telerehabilitation Versus Conventional Face-to-Face Rehabilitation After Cochlear Implantation: Prospective Intervention Pilot Study
Source: JMIR Rehabil Assist Technol. 2021 Mar 12;8(1):e20405. doi: 10.2196/20405 (PMC8082947; doi:10.2196/20405)
Supplement: Multimedia Appendix 7 [file rehab_v8i1e20405_app7.docx]

**Multimedia Appendix 7.** Bochum Usability Questionnaire; n=20 (100%) for each statement except in subtest “Videoconferencing” n=15 (100%).

| *For each of the following statements, please judge how true they are.* | | **Not true**  **0** | **1** | **2** | **3** | **Very true**  **4** | **Total**  **(%)** | **SD** |
| --- | --- | --- | --- | --- | --- | --- | --- | --- |
| **Introduction** | 1. The introduction videos were helpful. | 1 | 2 | 2 | 3 | 12 | 63 (90.0) | 1.26 |
|  | 1. The training tips were helpful. | 1 | 0 | 0 | 3 | 16 | 73 (91.3) | 0.94 |
| **Exercises** | 1. The exercises were relevant to everyday life. | 0 | 0 | 1 | 13 | 6 | 65  (81.3) | 0.55 |
|  | 1. The difficulty of the exercises was appropriate. | 1 | 0 | 2 | 4 | 13 | 68 (85.0) | 1.05 |
|  | 1. I understood the exercises very well. | 0 | 0 | 1 | 4 | 15 | 74  (92.3) | 0.58 |
|  | 1. I liked the inclusion of different speakers. | 0 | 0 | 3 | 3 | 14 | 71  (88.8) | 0.76 |
|  | 1. The speech rate was appropriate. | 0 | 1 | 1 | 4 | 14 | 71  (88.8) | 0.83 |
|  | 1. I got enough help. | 0 | 0 | 2 | 3 | 15 | 73  (91.3) | 0.68 |
|  | 1. I liked the concept of the journey. | 0 | 0 | 1 | 0 | 19 | 78  (97.5) | 0.45 |
| **Feedback** | 1. I have received sufficient feedback on my performance. | 0 | 0 | 1 | 3 | 16 | 75  (93.8) | 0.55 |
|  | 1. The feedback gave me sufficient feedback on my performance. | 0 | 0 | 0 | 6 | 14 | 74 (92.5) | 0.47 |
|  | 1. The feedback encouraged me to continue to practice. | 0 | 0 | 0 | 2 | 18 | 78 (97.5) | 0.31 |
| **Statistics** | 1. I looked at the statistics at least once a week. | 5 | 1 | 0 | 3 | 11 | 54  (67.5) | 1.75 |
|  | 1. The statistical evaluation helped me to assess my results. | 4 | 0 | 1 | 2 | 13 | 60 (75.0) | 1.63 |
|  | 1. The statistics were clear and understandable. | 2 | 0 | 1 | 3 | 14 | 67  (83.8) | 1.27 |
| **Video-conferencing** | 1. I could easily use the video conference. | 0 | 1 | 5 | 1 | 8 | 46  (76.7) | 1.10 |
|  | 1. The therapist's feedback was helpful. | 0 | 0 | 2 | 2 | 11 | 54 (90.0) | 0.74 |
|  | 1. I think the framework of the videoconference is as statisfying as the personal contact. | 1 | 2 | 2 | 2 | 8 | 44  (73.3) | 1.39 |
| **Design** | 1. I like the design of the digital training program. | 0 | 0 | 0 | 8 | 12 | 72 (90.0) | 0.51 |
|  | 1. The font size was appropriate. | 0 | 0 | 0 | 2 | 18 | 78  (97.5) | 0.31 |
|  | 1. The buttons were large enough. | 0 | 0 | 0 | 4 | 16 | 76 (95.0) | 0.41 |
| **Motivation** | 1. I enjoyed working with the program. | 0 | 0 | 0 | 4 | 16 | 76 (95.0) | 0.41 |
|  | 1. The digital training worked out well for me. | 0 | 1 | 2 | 4 | 13 | 69 (86.3) | 0.89 |
|  | 1. The digital training I allowed me to manage myself. | 1 | 0 | 2 | 6 | 11 | 66 (82.5) | 1.04 |
|  | 1. I felt tense when using the program. | 9 | 3 | 3 | 1 | 4 | 52 (65.0) | 1.61 |
| **Overall Assessment** | 1. The technology worked well. | 1 | 0 | 2 | 5 | 12 | 67  (83.8) | 1.04 |
|  | 1. Digital training is a suitable option for me. | 0 | 0 | 0 | 2 | 18 | 78  (97.5) | 0.31 |
|  | 1. Digital training made it easier for me to access to the therapy. | 0 | 1 | 3 | 1 | 15 | 70  (87.5) | 0.95 |
|  | 1. Digital training can be and an additive to training in clinic. | 2 | 0 | 1 | 1 | 16 | 69 (86.3) | 1.28 |
|  | 1. I would recommend digital training to other people. | 0 | 0 | 0 | 1 | 19 | 79 (98.8) | 0.23 |
|  | 1. Digital training motivated me to actively engage with my hearing problem. | 2 | 0 | 2 | 2 | 14 | 66 (82.5) | 1.31 |
|  | 1. My environment was interested in the training and supported me. | 6 | 0 | 2 | 1 | 10 | 51  (63.8) | 1.82 |
|  | 1. It was easy for me to stick to the number of training days (n=5) claimed per week. | 0 | 2 | 0 | 2 | 16 | 72 (90.0) | 0.94 |
|  | 1. It was easy for me to stick to the time claimed for training (25 min) per day. | 0 | 0 | 0 | 1 | 19 | 79 (98.8) | 0.23 |
