# Supplementary material for: Tablet-Based Telerehabilitation Versus Conventional Face-to-Face Rehabilitation After Cochlear Implantation: Prospective Intervention Pilot Study
Source: JMIR Rehabil Assist Technol. 2021 Mar 12;8(1):e20405. doi: 10.2196/20405 (PMC8082947; doi:10.2196/20405)
Supplement: Multimedia Appendix 8 [file rehab_v8i1e20405_app8.docx]

**Multimedia Appendix 8**. Results of subjective audiological self-rating based on the Oldenburg Inventory-R score; n=20 (100%) for each test and interval.

| **Topic** | **Time of testing** | **max** | **mean** | **SD** | **P** |
| --- | --- | --- | --- | --- | --- |
| **Listening in quiet** | T1 | 20 | 13.9 | 3.0 | .097 |
|  | T2 | 20 | 13.3 | 3.3 |  |
|  | T2 | 20 | 13.3 | 3.3 | .085 |
|  | T3 | 20 | 14.2 | 3.4 |  |
| **Listening in noise** | T1 | 20 | 8.5 | 4.0 | .0030** |
|  | T2 | 20 | 9.6 | 3.9 |  |
|  | T2 | 20 | 9.6 | 3.9 | .79 |
|  | T3 | 20 | 9.6 | 4.0 |  |
| **Localization** | T1 | 8 | 3.8 | 1.5 | .42 |
|  | T2 | 8 | 4.0 | 1.7 |  |
|  | T2 | 8 | 4.0 | 1.7 | .58 |
|  | T3 | 8 | 4.2 | 1.8 |  |
| **Listening effort** | T1 | 8 | 4.2 | 1.5 | .69 |
|  | T2 | 8 | 4.3 | 1.8 |  |
|  | T2 | 8 | 4.3 | 1.8 | .58 |
|  | T3 | 8 | 4.2 | 1.3 |  |
| **Others** | T1 | 16 | 9.5 | 2.6 | .39 |
|  | T2 | 16 | 9.9 | 2.9 |  |
|  | T2 | 16 | 9.9 | 2.9 | .55 |
|  | T3 | 16 | 10.0 | 2.9 |  |
| **Social interaction** | T1 | 100 | 40.9 | 21.3 | .17 |
|  | T2 | 100 | 38.5 | 22.7 |  |
|  | T2 | 100 | 38.5 | 22.7 | .21 |
|  | T3 | 100 | 34 | 21.6 |  |
| **Improvement in auditory skills (assessed only at T2 & T3)** | T2 | 16 | 11.2 | 2.7 | .34 |
|  | T3 | 16 | 11.9 | 2.5 |  |
