# Supplementary material for: Tablet-Based Telerehabilitation Versus Conventional Face-to-Face Rehabilitation After Cochlear Implantation: Prospective Intervention Pilot Study
Source: JMIR Rehabil Assist Technol. 2021 Mar 12;8(1):e20405. doi: 10.2196/20405 (PMC8082947; doi:10.2196/20405)
Supplement: Multimedia Appendix 9 [file rehab_v8i1e20405_app9.docx]

**Multimedia Appendix 9.** Overview of the costs during the 3-week intervention study. (A) Patient’s data. (B) Therapist’s data.

| A) Patient’s data | | |
| --- | --- | --- |
| Economic Aspect | FTF* | CBAT |
| Time spent on the road on average | 234 min (SD 58.6) | 0 |
| Travel distance on average | 240 km (SD 80.7) | 0 |

| B) Therapist’s data | | | | |
| --- | --- | --- | --- | --- |
| Economic Aspect | FTF time | FTF costs | CBAT time | CBAT costs |
| Duration of intervention  on average | 360 min | 210.00 Euro  US $ 256.20 | 60 min | 35.00 Euro  US $ 42.70 |
| Preparation and follow-up on average | 90 min | 52.50 Euro  US $ 64.20 | 30 min | 17.50 Euro  US $ 21.35 |
| Total time/costs | 450 min | 262.50 Euro  US $ 320.25 | 90 min | 52.50 Euro  US $ 64.20 |
| Total time/costs for the whole initial rehabilitation (20 x 120 min) | 3000 min | 1750.00 Euro  US $ 2135.00 | 600 min | 350.00 Euro  US $ 427.00 |

* Outward and return journey

FTF: face-to-face therapy, CBAT: Computer-based auditory training, costs for a therapist: 35 Euro (US $ 42,7) per hour.
